# The predictive value of coronary microvascular dysfunction for left ventricular reverse remodelling in dilated cardiomyopathy

**DOI:** 10.3389/fcvm.2023.1301509

**Published:** 2023-12-04

**Authors:** Ao Kan, Yinping Leng, Shuhao Li, Fang Lin, Qimin Fang, Xinwei Tao, Mengyao Hu, Lianggeng Gong

**Affiliations:** ^1^ Department of Radiology, The Second Affiliated Hospital of Nanchang University, Nanchang, China; ^2^ Department of Medical, Bayer Healthcare, Shanghai, China

**Keywords:** coronary microvascular dysfunction, myocardial perfusion, cardiac magnetic resonance, dilated cardiomyopathy, left ventricular reverse remodelling

## Abstract

**Aims:**

To evaluate the degree of coronary microvascular dysfunction (CMD) in dilated cardiomyopathy (DCM) patients by cardiac magnetic resonance (CMR) first-pass perfusion parameters and to examine the correlation between myocardial perfusion and left ventricle reverse remodelling (LVRR).

**Methods:**

In this study, 94 DCM patients and 35 healthy controls matched for age and sex were included. Myocardial perfusion parameters, including upslope, time to maximum signal intensity (Time_max_), maximum signal intensity (SI_max_), baseline signal intensity (SI_baseline_), and the difference between maximum and baseline signal intensity (SI_max−baseline_) were measured. Additionally, left ventricular (LV) structure, function parameters, and late gadolinium enhancement (LGE) were also recorded. The parameters were compared between healthy controls and DCM patients. Univariable and multivariable logistic regression analyses were used to determine the predictors of LVRR.

**Results:**

With a median follow-up period of 12 months [interquartile range (IQR), 8–13], 41 DCM patients (44%) achieved LVRR. Compared with healthy controls, DCM patients presented CMD with reduced upslope, SI_baseline_, and increased Time_max_ (all *p* < 0.01). Time_max_, SI_max_, and SI_max−baseline_ were further decreased in LVRR than non-LVRR group (Time_max_: 60.35 [IQR, 51.46–74.71] vs. 72.41 [IQR, 59.68–97.70], *p *= 0.017; SI_max_: 723.52 [IQR, 209.76–909.27] vs. 810.92 [IQR, 581.30–996.89], *p *= 0.049; SI_max−baseline_: 462.99 [IQR, 152.25–580.43] vs. 551.13 [IQR, 402.57–675.36], *p *= 0.038). In the analysis of multivariate logistic regression, Time_max_ [odds ratio (OR) 0.98; 95% confidence interval (CI) 0.95–1.00; *p *= 0.032)], heart rate (OR 1.04; 95% CI 1.01–1.08; *p *= 0.029), LV remodelling index (OR 1.73; 95% CI 1.06–3.00; *p *= 0.038) and LGE extent (OR 0.85; 95% CI 0.73–0.96; *p *= 0.021) were independent predictors of LVRR.

**Conclusions:**

CMD could be found in DCM patients and was more impaired in patients with non-LVRR than LVRR patients. Time_max_ at baseline was an independent predictor of LVRR in DCM.

## 1. Introduction

Dilated cardiomyopathy (DCM) is a common disease leading to heart failure and heart transplantation worldwide (1). It is defined by enlarged ventricles and systolic dysfunction not caused by abnormal loading conditions or coronary artery disease (1, 2). The coronary microvascular dysfunction (CMD) of DCM patients has been identified by various imaging techniques under both rest and stress conditions (3–7). Cardiac magnetic resonance (CMR) first-pass perfusion imaging can noninvasively qualitatively and semi-quantitatively assess CMD in DCM patients (3, 4, 8), and the diagnostic accuracy is similar to invasive angiography (9–11). CMD had incremental predicted value for poor prognosis in DCM patients over the degree of LV functional impairment (6, 12, 13).

With the improvement of heart failure treatment, more patients are experiencing left ventricle reverse remodelling (LVRR) with a favorable long-term prognosis (14–16). LVRR means the improvement of left ventricular ejection fraction (LVEF) accompanied by decreased left ventricular (LV) dimension (15). Prediction of LVRR plays a vital role in risk stratification and treatment strategies. With its high spatial and temporal resolution, cardiac magnetic resonance (CMR) can non-invasively and thoroughly evaluate cardiac structure, function, myocardial tissue characteristics, and myocardial perfusion in one stop. Several clinical and CMR parameters have been identified as indicators of LVRR in DCM, including lack of familial DCM history, TTN gene mutations, female, reduced LVEF, the ratio of the global longitudinal peak strain and the absence of late gadolinium enhancement (LGE), etc. (15, 17–21). Nevertheless, the relationship between myocardial perfusion and LVRR in patients with DCM hasn't been explored yet. Thus, our objectives were to (1) assess the differences of CMR parameters at the baseline parameters among healthy controls and DCM patients and (2) explore the independent predictors for LVRR in DCM patients.

## 2. Materials and methods

### 2.1. Study population

**Figure 1:**
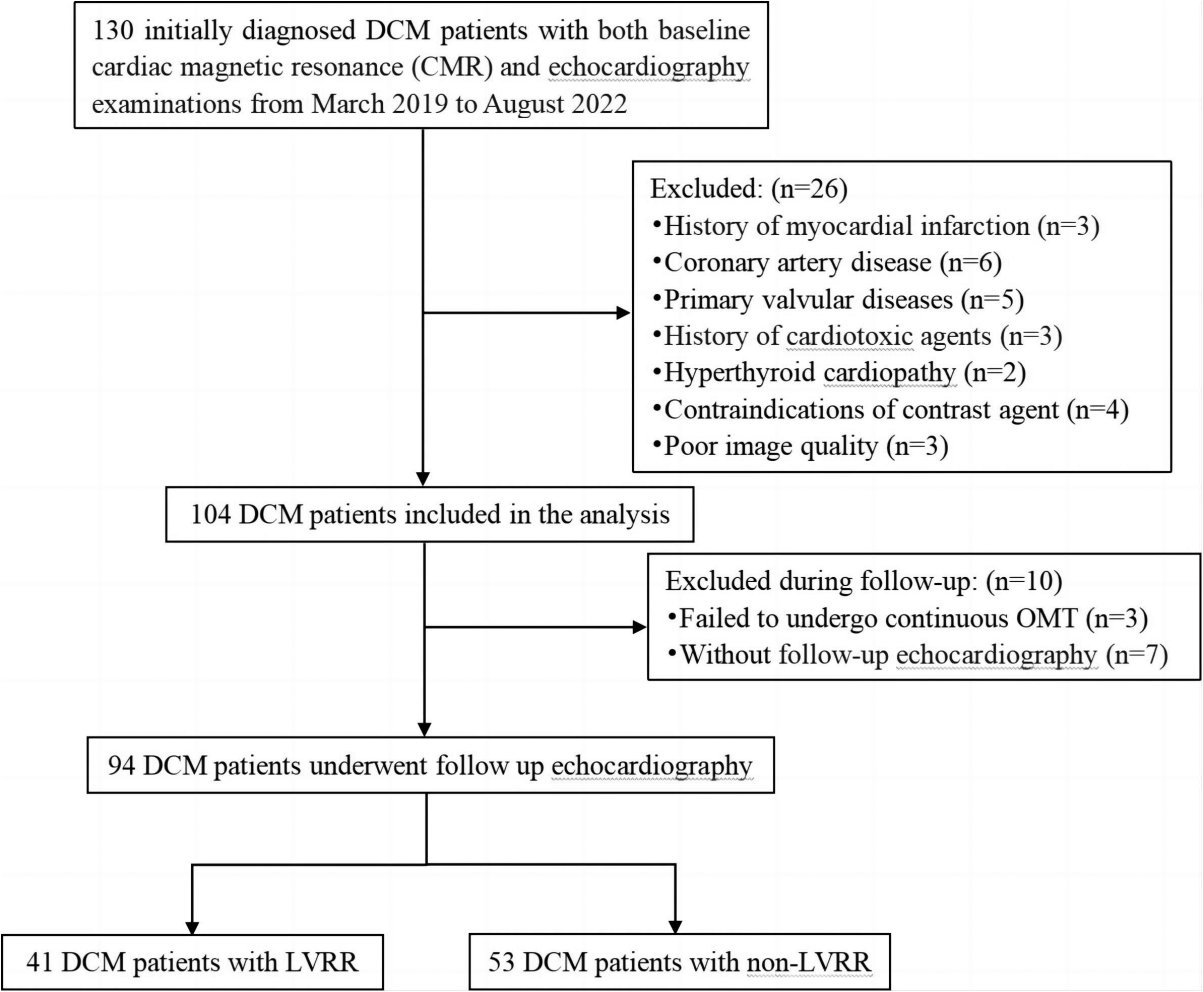
Flow diagram of the study.

A retrospective analysis was performed on initially diagnosed DCM patients who underwent baseline CMR and echocardiography examinations at our institution from March 2019 to August 2022. The time duration between baseline CMR and initial echocardiography was within 3 days. The criteria for inclusion in the study were as follows: reduced LVEF ≤45% and an increase in left ventricular end-diastolic dimension (LVEDD) ≥55 mm as determined by CMR (22). A total of 130 patients were initially enrolled in our study. The exclusion criteria were: (1) previous myocardial infarction (*n* = 3) or significant narrowing of the coronary arteries (>50%) determined by coronary artery computed tomography or coronary angiography (*n* = 6); (2) abnormal loading conditions due to other heart diseases (such as valvular disease, hyperthyroid cardiomyopathy, previous exposure to cardiotoxic agents) (*n* = 10); (3) contraindications of contrast agent (*n* = 4); (4) inadequate image quality (*n* = 3). Additionally, we eliminated individuals who didn't undergo follow-up echocardiography (*n* = 7), and those who were not administered continuous optimal medical therapies (OMT) (*n* = 3). Ultimately, 94 patients with DCM were enrolled in the study. The detailed flow diagram of this study is shown in Figure 1. We also included 35 age- and sex-matched healthy controls who underwent CMR as part of their health physical examination. These healthy controls had no cardiovascular diseases, chronic disease, or arrhythmia. Our institutional ethics review committee approved this retrospective study, and the requirement for informed consent was waived.

### 2.2. CMR imaging protocols

The CMR scans were obtained using a 3.0 T whole-body scanner (Discovery MR750W; GE Healthcare, Milwaukee, CA, USA) with a 30-element body phased-array coil. The acquisition process of all images included electrocardiogram (ECG) triggering and respiratory gating. CMR cine images of the short-axis, long-axis, 2-chamber, 3-chamber and 4-chamber views were obtained using steady-state free precession sequences, covering the entire range from the base to the apical level. The sequence parameters were as follows: repetition time (TR) of 3.9 ms, echo time (TE) of 1.6 ms, field of view (FOV) of 380 × 380 mm^2^, matrix size of 256 × 256 pixels, flip angle of 55° and a slice thickness of 6 mm. The dose of gadobutrol (Gadovist, Bayer Health Pharmaceuticals, Germany) was administered at a dosage of 0.1 mmol/kg. The injection was given intravenously at a rate of 3.0 ml/s, followed by a saline flush of 10–20 ml at the same rate. Concurrently the three standard short-axis slices in first-pass perfusion (basal, middle and apical slices) were obtained by fast gradient echo sequence (FGRET). The parameters of first-pass perfusion were: TR 7.0 ms; TE 1.6 ms; FOV 360 × 360 mm^2^; matrix 260 × 280 pixels; flip angle 20°; slice thickness 8 mm. LGE imaging was obtained using an inversion recovery gradient echo sequence approximately 10–15 min after contrast administration. The imaging parameters were: TR 4.8 ms; TE 1.6 ms; FOV 360 × 360 mm^2^; matrix 140 × 180 pixels; flip angle 20°; slice thickness 8 mm.

### 2.3. Image analysis

**Figure 2:**
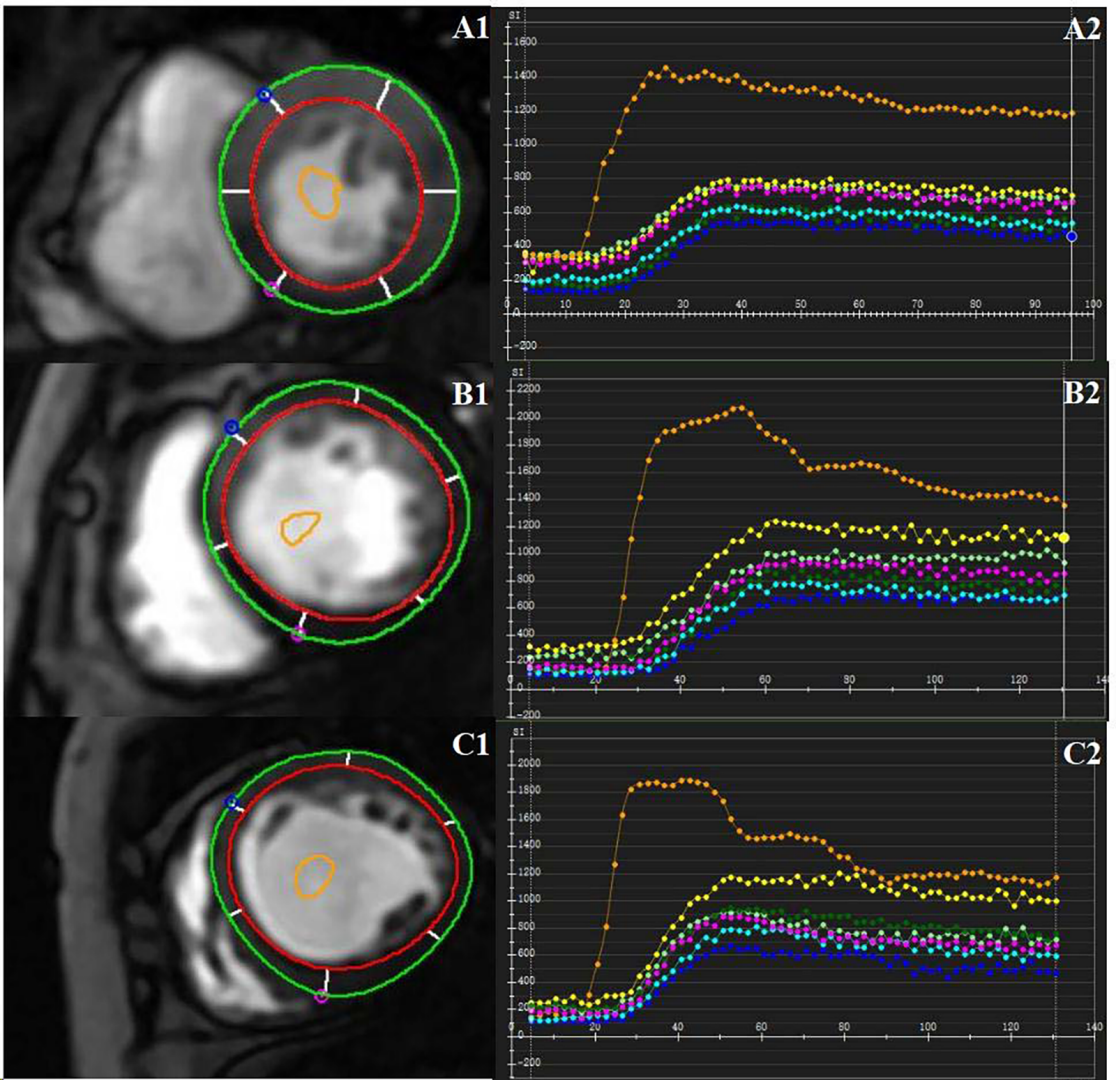
CMR-derived first-past perfusion imaging analysis. Manually delineate the epicardial and endocardial borders. A region of interest was drawn in the blood pool as a contrast. Representative first-pass myocardial perfusion images and the time-signal intensity curves of a healthy control subject **(A1**, **A2)**, one DCM patient without LVRR **(B1**, **B2)**, and one DCM patient with LVRR **(C1**, **C2)**.

Two experienced radiologists used CVI42 software (Circle Cardiovascular Imaging, Inc. Calgary, Canada) to conduct all image analyses. The endocardial and epicardial boundaries of the three short-axis slices were manually outlined on first-pass perfusion images, and a region of interest (ROI) was delineated within the blood pool to serve as a contrast. The time-signal intensity curves of 16 myocardial segments (Bull's eye plot) (23) for both myocardial and blood pool were acquired, excluding the apex, due to significant measurement inaccuracies. The semi-quantitative perfusion parameters, including upslope, time to maximum signal intensity (Time_max_), maximum signal intensity (SI_max_), baseline signal intensity (SI_baseline_), and the difference between maximum and baseline signal intensity (SI_max−baseline_), were automatically derived from the myocardial time-signal intensity curves (Figure 2). This study used the average value of 16 segments to calculate the corresponding global perfusion value.

Manually, the optimal end-diastole and end-systole borders of the LV endocardium and epicardium in cine images were also outlined. The contour of the endocardium did not encompass the papillary muscles and trabeculations. From the short-axis cine images, cardiac geometry and function parameters were obtained, which included the index of LV end-diastolic and end-systolic volume (LVEDVi, LVESVi), the index of left ventricular mass (LVMi), LVEF and the index of LV stroke volume (LVSVi). The values were scaled to body surface area (BSA) for indexing. Furthermore, the LV remodelling index (LVRI) was determined by dividing the LV mass by the LV end-diastolic volume (LVEDV). Using CVI42's tissue tracking module, the LV global peak strain (GPS) was measured from the long- and short-axis cine images. The symbols for positive and negative of GPS indicate distinct motion directions.

LGE was considered to be present when it was observed in both long- and short-axis planes, and the extent surpasses the localized ventricular insertion sites (24). Two independently experienced operators confirmed LGE presence and a third experienced operator provided adjudication. Using CVI42's tissue characteristic module, the two operators quantified the extent of LGE on short-axis LGE images using a threshold of 5 SD. The extent of LGE is indicated as a proportion of the left ventricular mass (25).

### 2.4. Follow-up and LVRR definition

The patients included in the study received continuous OMT according to the guidelines for treating patients with heart failure (HF) (26). Experienced sonographers, by the guidelines of the American Society of Echocardiography (27), conducted baseline and follow-up two-dimensional transthoracic echocardiography using a commercially accessible echocardiography system (General Electric Vivid-E95). The modified Simpson's method was used to evaluate LVEF. The parasternal long-axis perspective was utilized to measure LVEDD. LVRR was characterized as a significant increase in LVEF of at least 10% to a final value exceeding 35%, accompanied by a reduction in LVEDD of at least 10% compared to the initial echocardiography (15, 28).

### 2.5. Reproducibility analysis

Both inter- and intraobserver reproducibility was assessed for the global perfusion parameters, global strain parameters, and LGE extent in randomly selected 40 patients. To assess the intraobserver reproducibility, the same observer measured the CMR parameters twice, with a one-month interval. A second independent observer measured the values to assess intra-observer reproducibility. Both observers were unaware of the results of each other and the patients' medical records.

### 2.6. Statistical analysis

Mean ± SD was used to represent continuous variables with normal distribution, while medians with interquartile range (IQR) were used for variables with non-normal distribution. Frequencies and percentages were used to express categorical variables. The normality of distribution was tested using the Shapiro–Wilk test. A one-way analysis of variance (ANOVA) or Kruskal–Wallis test was used to compare continuous variables in the non-LVRR group, LVRR group, and healthy controls, depending on the normality of the data. Continuous variables between the DCM groups were compared using either an independent t-test or a Mann–Whitney *U* test. In contrast, categorical variables were compared using chi-square test or Fisher's exact test. As an exploratory study, Bonferroni post-hoc correction for multiple group comparison was not performed (21). The findings for analyses should be interpreted as exploratory. Predictors of LVRR were determined using univariable and multivariable logistic regression analyses. The multivariable logistic regression analysis included variables with a *p*-value < 0.05 in the univariable analysis without collinearity to determine the independent factors of LVRR. Bayesian information criterion (BIC) was used to avoid overfitting. Receiver operating characteristic (ROC) curves were employed to determine the area under the curve (AUC), sensitivity, specificity, positive predictive value (PPV) and negative predictive value (NPV) to quantify the predictive capability of the significant univariables and final multivariable regression model. We used the Spearman rank correlation to evaluate the correlation between perfusion parameters and LV geometry, function, strain, and LGE extent. The reproducibility of inter- and intraobserver of LV global perfusion, strain, and LGE extent were evaluated using the intraclass correlation coefficient (ICC). Two-sided *p *< 0.05 was attributed to statistical significance. The statistical analyses were performed using RStudio 4.1.2 and SPSS 26.0.

## 3. Results

### 3.1. Baseline clinical characteristics

Ninety-four patients were finally included with DCM, including 53 non-LVRR patients [mean age: 49.30 ± 14.58 years old; 58.49% (31/53) were male] and 41 LVRR patients [mean age: 45.95 ± 14.39 years old; 65.85% (27/41) were male]. The mean age of 35 healthy controls was 51.69 ± 13.36 years old, and 71.43% (25/35) were male. The median duration between the initial and subsequent echocardiography examinations was 12 [IQR, 8–13] months. The patients' baseline characteristics are displayed in Table 1. The healthy controls had lower HbA1c, higher HDL, and eGFR than DCM groups (*p *< 0.05 for all). Most DCM patients (61.70%) exhibited New York Heart Association (NYHA) class III or IV. The LVRR group had higher heart rate than healthy controls, and non-LVRR group (69.00 [IQR, 64.00–71.50] vs. 81.00 [IQR, 73.00–92.42], *p *< 0.001; 72.00 [IQR, 67.00–85.00] vs. 81.00 [IQR, 73.00–92.42], *p *= 0.014). The other clinical parameters had no significant statistical differences between the two DCM groups.

**Table 1:** Baseline characteristics of the healthy controls and DCM patients.

	Healthy controls (*n* = 35)	DCM (*n* = 94)		*P* value (non-LVRR vs. LVRR)
		Non-LVRR (*n* = 53)	LVRR (*n* = 41)	
Clinical parameters				
Age (year)	51.69 (13.36)	49.30 (14.58)	45.95 (14.39)	0.495
Males, *n* (%)	25 (71.43%)	31 (58.49%)	27 (65.85%)	0.784
BSA (m^2^)	1.77 [1.58; 1.85]	1.70 [1.58; 1.82]	1.78 [1.63; 1.87]	0.384
SBP (mmHg)	127.00 [116.00; 145.00]	120.00 [106.00; 133.00]	122.00 [110.00; 133.00]	0.703
DBP (mmHg)	80.00 [68.50; 90.50]	77.00 [68.00; 88.00]	78.00 [66.00; 86.00]	0.945
Heart rate (beat/min)	69.00 [64.00; 71.50]	72.00 [67.00; 85.00]*	81.00 [73.00; 92.42]**^#^**	**0**.**014**
HF duration (day)	–	20.00 [4.00; 57.00]	30.00 [4.50; 60.00]	0.604
Familial history of DCM, *n* (%)		4 (7.55%)	3 (7.32%)	0.99
NYHA III/IV, *n* (%)	–	32 (60.38%)	26 (63.41%)	0.931
Comorbidity, *n* (%)				
Hypertension	–	14 (26.42%)	7 (17.07%)	0.407
Diabetes	–	6 (11.32%)	4 (9.76%)	0.990
Dyslipidemia	–	23 (43.40%)	19 (46.34%)	0.940
Laboratory examination				
BNP (pg/ml)	–	904.44 [350.00; 1,870.60]	700.66 [311.00; 1,179.32]	0.302
HCT (%)	42.04 (4.39)	43.16 (5.24)	43.24 (4.97)	0.997
HbA1c (%)	5.10 [4.15; 5.45]	5.70 [5.40; 6.00]*	5.70 [5.40; 5.92]**^#^**	0.933
TG (mmol/L)	1.18 [0.77; 1.89]	1.08 [0.84; 1.46]	1.30 [1.01; 1.95]	0.081
TC (mmol/L)	4.31 (1.17)	4.31 (0.95)	4.50 (1.14)	0.665
HDL (mmol/L)	1.30 [1.07; 1.42]	1.01 [0.78; 1.33]*	0.95 [0.77; 1.25]**^#^**	0.547
LDL (mmol/L)	2.37 (0.82)	2.58 (0.80)	2.82 (0.80)	0.346
eGFR (ml/min/1.73 m^2^)	99.21 (10.68)	79.49 (23.17)*	85.01 (21.78)**^#^**	0.386
Medical treatment, *n* (%)				
ARNi	–	29 (54.72%)	20 (48.78%)	0.716
ACEI/ARB	–	21 (39.62%)	21 (51.22%)	0.362
Beta blocker	–	53 (100.00%)	41 (100.00%)	0.990
MRA	–	34 (64.15%)	38 (92.68%)	**0**.**003**
Diuretics	–	46 (86.79%)	28 (68.29%)	0.055
SGLT2i	–	11 (20.75%)	9 (21.95%)	0.990

All values are presented as mean ± SD or median [Q1–Q3] or *n* (%). Bold values indicate significant *p* < 0.05.

DCM, dilated cardiomyopathy; LVRR, left ventricular reverse remodeling; BSA, body surface area; SBP, systolic blood pressure; DBP, diastolic blood pressure; HF, heart failure; NYHA, New York Heart Association; BNP, brain natriuretic peptide; HCT, hematocrit; HbA1c, glycated hemoglobin; TG, triglycerides; TC, total cholesterol; HDL, high-density lipoprotein; LDL, low-density lipoprotein; eGFR, estimated glomerular filtration rate; ARNi, angiotensin receptor-neprilysin inhibitors; ACEI, angiotensin-converting enzyme inhibitor; ARB, angiotensin receptor blocker; MRA, mineralocorticoid receptor antagonist; SGLT2i, sodium-glucose cotransporter-2 inhibitors.

* Non-LVRR group versus Healthy controls, *p* < 0.05.

# LVRR group versus Healthy controls, *p* < 0.05.

### 3.2. Comparison of CMR findings among the three groups

**Figure 3:**
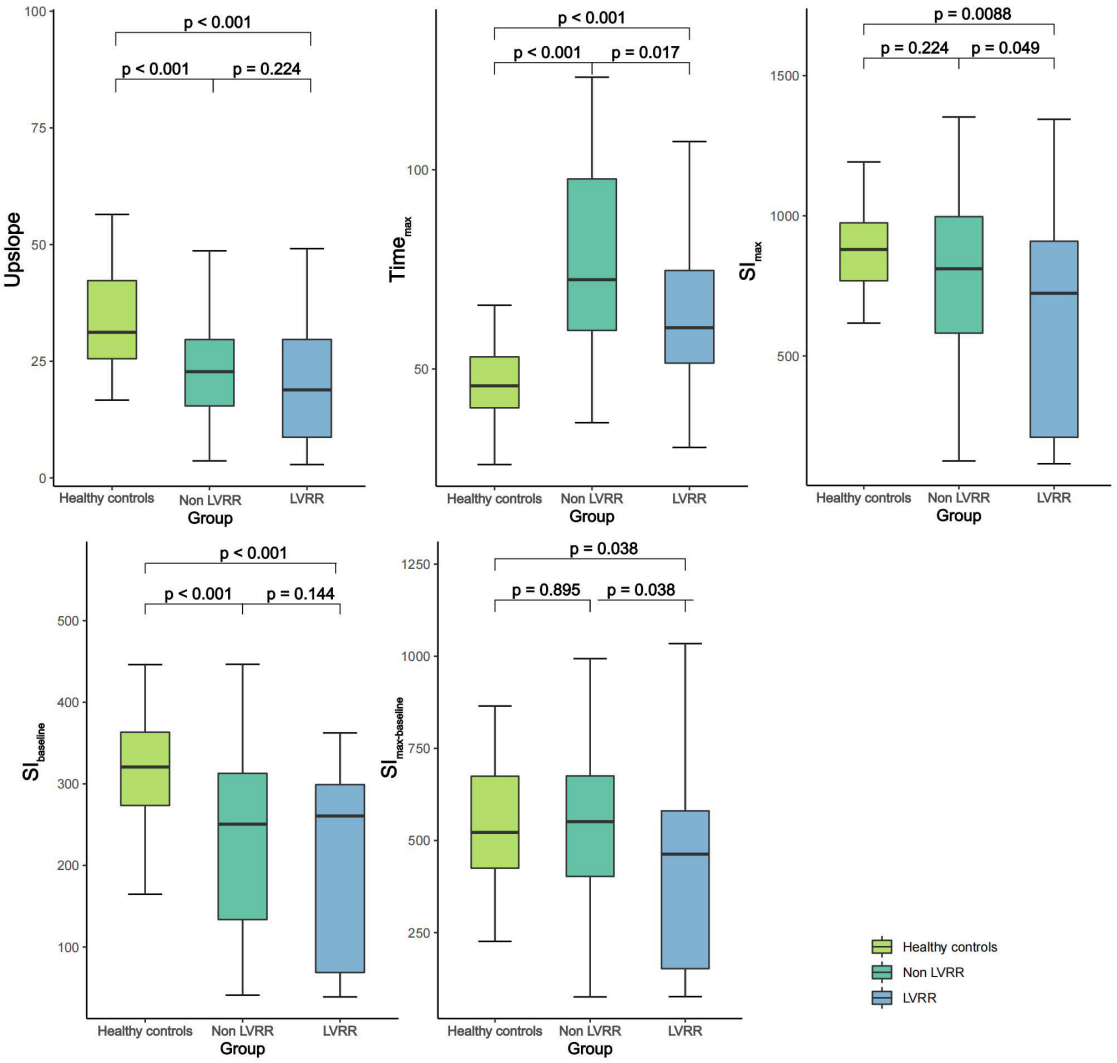
Comparison of first-pass perfusion parameters among healthy control group and DCM patients with and without LVRR. DCM, dilated cardiomyopathy; LVRR, left ventricular reverse remodeling; Time_max_, time to maximum signal intensity; SI_max_, maximum signal intensity; SI_baseline_, baseline signal intensity; SI_max−baseline_, the difference between maximum and baseline signal intensity.

**Figure 4:**
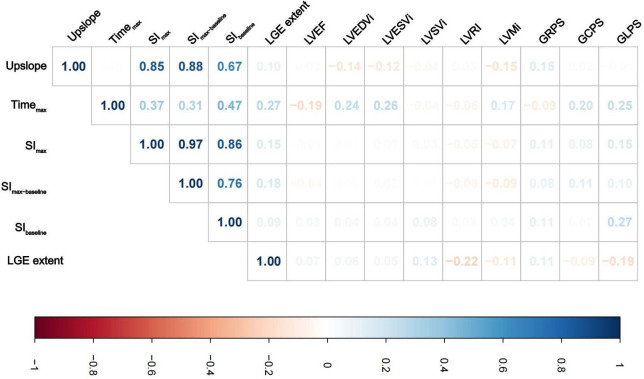
Correlations analysis between myocardial perfusion and LV geometry parameters, function parameters, global peak strain, and LGE extent in DCM patients. Colors represent the correlation coefficients, and its intensity represents the coefficient's value (**A**) Abbreviations are as in Table 2.

Table 2 presents the LV perfusion parameters, structure, function and strain of the two groups of DCM patients. The healthy controls had better LV function, higher LVRI and GPS, and lower LV geometry parameters than the DCM groups (*p *< 0.05 for all). Except for SI_max_ and SI_max−baseline_ of non-LVRR patients, the two DCM groups had significantly worse myocardial perfusion compared with healthy controls (*p *< 0.001 for all) (Figure 3). At baseline, compared to non-LVRR patients, the LVRR patients exhibited a notable reduction in geometry parameters while LVRI increased. Furthermore, the LVRR group exhibited lower perfusion parameters (Time_max_, SI_max,_ and SI_max−baseline_) compared to the non-LVRR group (Time_max_: 60.35 [IQR, 51.46–74.71] vs. 72.41 [IQR, 59.68–97.70], *p *= 0.017; SI_max_: 723.52 [IQR, 209.76–909.27] vs. 810.92 [IQR, 581.30–996.89], *p *= 0.049; SI_max−baseline_: 462.99 [IQR, 152.25–580.43] vs. 551.13 [IQR, 402.57–675.36], *p *= 0.038). Other perfusion parameters, LV function parameters, and GPS values were not significantly different between the LVRR and non-LVRR groups. The non-LVRR group had more LGE and a more significant extent than the LVRR group (79.25% vs. 56.10%, *p *= 0.029; 0.50 [IQR, 0.00–3.55] vs. 3.41 [IQR, 0.97–6.92], *p *< 0.001). Time_max_ and LVEF had a weak correlation (*r* = −0.19, *p *= 0.047). The upslope, SI_max_, and SI_max−baseline_ were weakly correlated with LVMi (*r* = −0.15, *p *= 0.021; *r* = −0.07, *p *= 0.047; *r* = −0.09, *p *= 0.047) (Figure 4).

**Table 2:** Comparison of CMR parameters among DCM patients with and without LVRR.

	Healthy controls (*n* = 35)	DCM		*P* value
		Non-LVRR (*n* = 53)	LVRR (*n* = 41)	(Non-LVRR vs. LVRR)
LV geometry and function				
LVEF (%)	52.22 [48.83; 55.90]	13.67 [11.36; 19.84]*	16.05 [11.27; 21.71]**^#^**	0.309
LVEDVi (ml/m2)	71.86 [57.32; 80.19]	172.31 [145.77; 190.64]*	149.56 [114.11; 179.89]**^#^**	**0**.**014**
LVESVi (ml/m2)	34.87 [25.19; 40.90]	140.57 [121.70; 166.32]*	120.96 [89.13; 158.61]**^#^**	**0**.**015**
LVSVi (ml/m2)	36.91 [30.23; 41.13]	25.89 [19.48; 32.90]*	22.76 [18.66;28.79]**^#^**	0.221
LVMi (g/m2)	46.66 [38.12; 51.69]	69.53 [56.84; 88.37]*	70.45 [55.84; 83.94]**^#^**	0.601
LVRI (g/ml)	0.67 [0.53; 0.73]	0.42 [0.36; 0.49]*	0.47 [0.42; 0.56]**^#^**	**0**.**011**
Global perfusion parameters				
Upslope	31.20 [25.54; 42.28]	22.77 [15.42; 29.66]*	18.86 [8.72; 29.67]**^#^**	0.244
Timemax	45.77 [40.24; 53.04]	72.41 [59.68; 97.70]*	60.35 [51.46; 74.71]**^#^**	**0**.**017**
SImax	879.72 [768.11; 974.76]	810.92 [581.30; 996.89]	723.52 [209.76; 909.27]**^#^**	**0**.**049**
SIbaseline	320.66 [273.48; 363.32]	250.57 [133.68; 312.87]*	260.62 [68.76; 299.05]**^#^**	0.144
SImax−baseline	522.01 [425.02; 674.53]	551.13 [402.57; 675.36]	462.99 [152.25; 580.43]**^#^**	**0**.**038**
LV GPS (%)				
GRPS	31.29 [26.98; 40.14]	9.21 [6.22; 11.88]*	7.69 [6.58;10.48]**^#^**	0.323
GCPS	−18.92 [−20.30; −18.59]	−5.71 [−7.70; −4.29]*	−6.59 [−8.82;−4.72]**^#^**	0.172
GLPS	−12.26 [−14.18; −10.34]	−4.78 [−5.80; −2.83]*	−4.76 [−5.76;−3.77]**^#^**	0.678
LGE				
LGE presence, *n* (%)	–	42 (79.25%)	23 (56.10%)	**0**.**029**
LGE extent, (%)	–	3.41 [0.97; 8.79]	0.50 [0.00; 3.55]	**<0**.**001**

All values are presented as median [Q1–Q3] or *n* (%). “−” indicates the direction of strains. Bold values indicate significant *p* < 0.05.

DCM, dilated cardiomyopathy; LVRR, left ventricular reverse remodeling; LV, left ventricular; LVEF, left ventricular ejection fraction; LVEDVi, left ventricular end-diastolic volume index; LVESVi, left ventricular end-systolic volume index; LVSVi, left ventricular stroke-volume index; LVMi, left ventricular mass index; LVRI, left ventricular remodeling index; Time_max_, _ _time to maximum signal intensity; SI_max_, maximum signal intensity; SI_baseline_, baseline signal intensity; SI_max−baseline_, the difference between maximum and baseline signal intensity; GPS, global peak strain; GRPS, global radial peak strain; GCPS, global circumferential peak strain; GLPS, global longitudinal peak strain; LGE, late gadolinium enhancement.

* Non-LVRR group versus Healthy controls, *p* < 0.05.

# LVRR group versus Healthy controls, *p* < 0.05.

### 3.3. Predictors of LVRR

**Figure 5:**
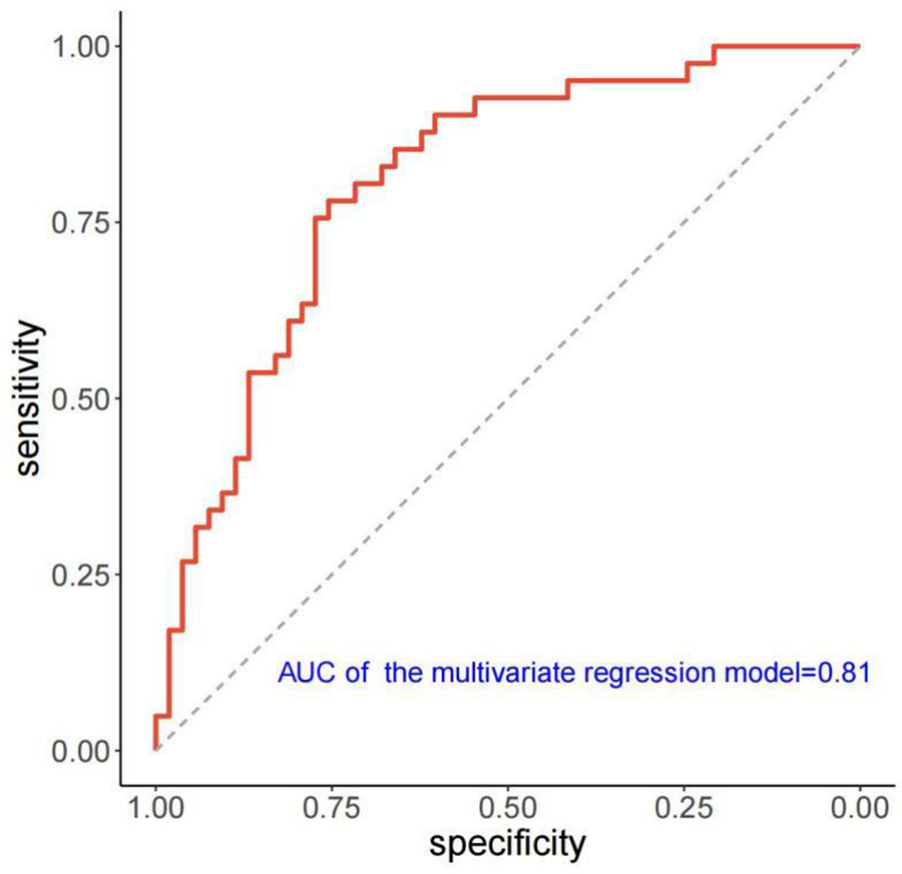
ROC curve of the multivariate regression model for predicting LVRR. ROC, receiver operating characteristic; AUC, Area under the curve; LVRR, left ventricular reverse remodeling.

Table 3 presents the results of univariate and multivariate logistic analyses conducted to evaluate the predictors of LVRR. Accroding to the univariate analysis, significant predictors of LVRR included heart rate, Time_max_, SI_max_, SI_max−baseline_, LVEDVi, LVRI, presence of LGE, and extent of LGE at baseline. LVEDVi was excluded due to collinearity, and SI_max_, SI_max−baseline_, and LGE presence were excluded by BIC. Finally, heart rate (OR 1.042; 95% CI 1.010–1.080; *p *= 0.029), Time_max_ (OR 0.975; 95% CI 0.952–0.997; *p *= 0.032), LVRI (OR 1.725; 95% CI 1.056–2.995; *p *= 0.038) and LGE extent (OR 0.850; 95% CI 0.730–0.956; *p *= 0.021) were independent predictors of LVRR. The AUC of the multivariate model yielded 0.81 (95% CI, 0.71–0.88) (Figure 5). The other predictive performance of the significant univariables and final multivariable regression model were shown in Table 4.

**Table 3:** Univariate and multivariate logistic regression analysis to predict LVRR.

	Univariate analysis		Multivariate analysis	
	OR (95% CI)	*P* value	OR (95% CI)	P value
Clinical and laboratory parameters				
Age, per 1-year	0.984 (0.956–1.012)	0.267		
Males	1.369 (0.588–3.188)	0.467		
SBP, per 10-mmHg	1.050 (0.823–1.339)	0.693		
DBP, per 10-mmHg	0.957 (0.701–1.305)	0.780		
Heart rate (beat/min)	1.039 (1.006–1.072)	**0** **.** **018**	1.042 (1.010–1.080)	0.029
NYHA III/IV	1.137 (0.491–2.637)	0.764		
BNP, per 1-pg/ml	1.000 (0.999–1.000)	0.402		
Global perfusion parameters				
Upslope	0.985 (0.952–1.020)	0.406		
Timemax	0.976 (0.957–0.995)	**0**.**014**	0.975 (0.952–0.997)	0.032
SImax	0.999 (0.997–1.000)	**0**.**025**		
SIbaseline	0.997 (0.994–1.001)	0.130		
SImax−baseline	0.998 (0.996–1.000)	**0**.**018**		** **
LV geometry and function				
LVEF, per SD	1.191 (0.789–1.798)	0.405		
LVEDVi, per SD	0.602 (0.371–0.978)	**0**.**040**	** **	** **
LVESVi, per SD	0.634 (0.4–1.004)	0.052		
LVSVi, per SD	0.694 (0.43–1.121)	0.135		
LVMi, per SD	0.829 (0.54–1.271)	0.389		
LVRI, per SD	1.798 (1.138–2.841)	**0**.**012**	1.725 (1.056–2.995)	0.038
LV GPS				
GRPS, per SD	0.736 (0.480–1.129)	0.160		
GCPS, per SD	0.746 (0.491–1.133)	0.169		
GLPS, per SD	0.851 (0.560–1.293)	0.449		
LGE				
LGE presence	0.335 (0.135–0.828)	**0**.**018**		
LGE extent	0.798 (0.687–0.926)	**0**.**003**	0.850 (0.730–0.956)	0.021

Bold values indicate significant *p* < 0.05.

CI, confidential interval; SD, standard deviation; Other abbreviations as in Table 2.

**Table 4:** The predictive performance of the univariate and multivariate of the muti-logistic regression.

	AUC (95% CI)	Sensitivity	Specificity	PPV	NPV
Heart rate	0.648 (0.537–0.759)	0.756 (0.625–0.888)	0.509 (0.375–0.644)	0.544 (0.415–0.673)	0.730 (0.587–0.873)
Time_max_	0.643 (0.531–0.756)	0.390 (0.241–0.540)	0.340 (0.212–0.467)	0.314 (0.186–0.441)	0.419 (0.271–0.566)
SI_max_	0.629 (0.514–0.743)	0.390 (0.241–0.540)	0.849 (0.753–0.945)	0.667 (0.478–0.855)	0.643 (0.531–0.755)
SI_max−baseline_	0.635 (0.520–0.750)	0.415 (0.264–0.565)	0.849 (0.753–0.945)	0.680 (0.497–0.863)	0.652 (0.540–0.765)
LVEDVi	0.648 (0.530–0.766)	0.415 (0.264–0.565)	0.925 (0.853–0.996)	0.810 (0.642–0.977)	0.671 (0.563–0.779)
LVRI	0.653 (0.540–0.765)	0.732 (0.596–0.867)	0.623 (0.492–0.753)	0.600 (0.464–0.736)	0.750 (0.622–0.878)
LGE extent	0.711 (0.609–0.812)	0.073 (0.00–0.153)	0.566 (0.433–0.699)	0.115 (0.00–0.238)	0.441 (0.323–0.559)
LGE presence	0.615 (0.521–0.710)	0.561 (0.409–0.713)	0.208 (0.098–0.317)	0.354 (0.238–0.470)	0.379 (0.203–0.556)
Multi-logistic model	0.807 (0.719–0.894)	0.902 (0.812–0.993)	0.604 (0.472–0.735)	0.638 (0.514–0.762)	0.889 (0.786–0.992)

AUC, area under the curve; CI, confidential interval; PPV, positive predictive value; NPV, negative predictive value; Other abbreviations as in Table 2.

### 3.4. Inter- and intraobserver variability of CMR perfusion, strain parameters and LGE extent

Table 5 shows the results of the inter- and intraobserver analyses of CMR perfusion parameters, LV global strain parameters, and LGE extent. The interobserver ICCs varied between 0.823 and 0.983, while the intraobserver ICCs varied between 0.821 and 0.993 for LV myocardial perfusion, strain parameters, and the LGE extent, indicating exceptional levels of reliability.

**Table 5:** Intra- and interobserver variabilities of LV myocardial strain.

	Intraobserver		Interobserver	
	ICC	95% CI	ICC	95% CI
Global perfusion parameters				
Upslope	0.983	0.968–0.991	0.993	0.987–0.996
Time_max_	0.823	0.665–0.906	0.832	0.683–0.911
SI_max_	0.954	0.914–0.976	0.962	0.929–0.980
SI_baseline_	0.831	0.680–0.910	0.821	0.662–0.905
SI_max−baseline_	0.925	0.859–0.961	0.938	0.883–0.967
LV GPS (%)				
GRPS	0.931	0.869–0.963	0.945	0.895–0.971
GCPS	0.967	0.938–0.983	0.987	0.975–0.993
GLPS	0.978	0.959–0.989	0.990	0.981–0.995
LGE				
LGE extent	0.953	0.902–0.978	0.971	0.939–0.986

ICC, intraclass correlation coefficient; Other abbreviations as in Table 2.

## 4. Discussion

Our study findings are as following: (1) The DCM patients with and without LVRR both had CMD; (2) Perfusion parameters in DCM patients were correlated with LVEF and LVMi; (3) Heart rate, Time_max_, LVRI, and LGE extent were independent predictors for LVRR in DCM patients.

With advanced technology, CMR myocardial perfusion imaging can non-invasively assess myocardial microcirculation without radiation. In DCM patients with the absence of coronary arterial disease, myocardial perfusion impairment indicates underlying abnormal function and structure of the coronary microcirculation, which causes CMD (29–31). Bietenbeck et al. found DCM patients had both lower MBF at rest and stress by first-pass based myocardial perfusion reserve (4), which was in line with the lower upslope, SI_baseline_ value and increased Time_max_ value at rest in our study. On the other hand, Gulati et al. found DCM patients had global higher rest MBF but significantly lower stress MBF and myocardial perfusion reserve (MPR) by CMR hybrid echo planar imaging sequence (3). The following reasons may explain the different in rest MBF: (1) The sample sizes of these studies were relatively small, and the presence ratio of LGE in DCM patients was higher both in Bietenbeck's (4) and our study. Since LGE is related to rest myocardial microcirculation (32, 33), fibrosis may impact the rest MBF; (2) The imaging sequences of myocardial perfusion were different, and the consistency between the two sequences needs further exploration. In addition, the LVRR group exhibited significantly reduced values of Time_max,_ SI_max_, and SI_max−baseline_ compared to the non-LVRR group. It implied non-LVRR group had more severe CMD.

In DCM patients, reduced stress MBF is associated with the degree of LV dysfunction and LVMi (3). Consistent with Gulati's results, we found that patients with DCM exhibited higher LVMi than healthy controls and negatively correlated with first-pass myocardial perfusion parameters. The possible mechanism is that vasodilatory capacity and density of coronary resistance vessels could not adapt to the increased myocardial mass in DCM. In return, long-term myocardial hypoperfusion results in fibrosis and adverse remodelling in DCM (29). Furthermore, our study, observed a negative correlation between Time_max_, defined as the duration from the start of the contrast agent to reach the highest signal intensity of myocardium (34), and LVEF. The increased Time_max_ may be attributed to the presence of CMD, which leads to a deterioration in systolic function and an extended wash-in time in DCM myocardium. Gulati et al. also verified the strong association between myocardial perfusion and systolic function in DCM patients, in which LVEF was an independent predictor of stress MBF (3).

Due to its close relationship with clinical outcomes, LVRR has become an essential target in clinical management at a cellular and molecular level. It can affect all components of cardiac tissue, including myocardial microcirculation (35). Isolated aortic stenosis patients have impaired MPR and stress MBF, quantified on ammonia N13 PET imaging, and were associated with adverse LV remodelling (36). After cardiac resynchronization therapy, LVRR was observed in non-ischemic cardiomyopathy patients with left bundle branch block, which was correlated with the improvement of LV septal perfusion (37). This suggests a potential correlation between myocardial perfusion and ventricular remodelling in non-ischemic cardiomyopathy. However, few studies have explored the correlation between myocardial perfusion and LVRR after OMT in DCM patients. We found that Time_max_ was an independent predictor for LVRR in DCM patients after LV function and structure adjustment. Higher Time_max_ indicates a longer wash-in time in damaged myocardium caused by severe CMD. The coronary microcirculation alterations in DCM include functional (a severe resistance microvessels dysfunction) and structural (microvessel density decrease, the remodelling, and obstruction of the lumen, etc.) (30, 38, 39). The role of the CMD in the progression of heart failure has been confirmed through endomyocardial biopsy of patients with DCM and animal models (30, 31). Thus, the aggravated CMD can result in progressive ventricular functional deterioration and adverse remodelling, possibly hindering LVRR after OMT treatment.

Apart from CMD parameters, heart rate, LVRI, and LGE extent were independent predictors for LVRR of DCM patients. The pathological changes of DCM included increased myocardial mass and dilated LV chamber size caused by irregular myocyte hypertrophy, damage, and myocardial interstitial fibrosis. Eccentric remodelling occurs in DCM patients when wall-thickening cannot balance the excessive volume overload and gradual chamber enlargement (40). The decreased cardiac output is commonly observed in DCM patients and can be compensated by increased heart rate, which positively impacts LVRR. At baseline the patients of LVRR group had smaller extent and less frequency of LGE. What's more, LGE extent could independently predict LVRR, which was in line with previous studies (14, 20, 41, 42). These findings suggested that the replacement myocardial fibrosis detected by the LGE technique played an important role in developing LVRR in DCM patients.

In summary, LVRR results from a complex interplay between myocardial perfusion, tissue characteristics, and cardiac function. Our study was the first to show the important predictive value of CMD for LVRR in DCM patients. Whether early treatment to improve myocardial microcirculation can promote the occurrence of LVRR in DCM patients and improve the prognosis of patients needs further research.

## 5. Limitations

Our study had several limitations. First, although we demonstrated the reproducibility of the semi-quantitative perfusion parameters derived from first-pass perfusion imaging, these results are limited in their generalizability because they vary greatly with various scanning sequences, imaging parameters, equipment, etc. Second, the study's median follow-up duration was 12 months. In order to validate the study's findings, a longer follow-up period is required. Third, this retrospective study was conducted in a single center with a relatively small sample size. Thus, selection bias might be present in this study. The findings of our research need to be validated by larger-scale and prospective studies.

## 6. Conclusions

Coronary microvascular dysfunction is present in DCM patients, and its severity is associated with the degree of LV impairment and LVMi. Time_max_, heart rate, LVRI, and LGE extent were independent predictors for LVRR in DCM patients.

## Data Availability

The raw data supporting the conclusions of this article will be made available by the authors, without undue reservation.
